# Safety of delivering bronchial thermoplasty in two treatment sessions

**DOI:** 10.1186/s12931-021-01901-x

**Published:** 2021-11-29

**Authors:** Kavya Koshy, Joy Sha, Kim Bennetts, David Langton

**Affiliations:** 1 Department of Thoracic Medicine, Frankston Hospital, Peninsula Health, 2 Hastings Road, Frankston, VIC 3199 Australia; 2 Faculty of Medicine, Nursing and Health Sciences, Monash University, Clayton, VIC Australia

**Keywords:** Asthma, Bronchial thermoplasty

## Abstract

**Background:**

Bronchial thermoplasty (BT) is a novel endoscopic therapy for severe asthma. Traditionally it is performed in three separate treatment sessions, targeting different portions of the lung, and each requires an anaesthetic and hospital admission. Compression of treatment into 2 sessions would present a more convenient alternative for patients. In this prospective observational study, the safety of compressing BT into two treatment sessions was compared with the traditional 3 treatment approach.

**Methods:**

Sixteen patients meeting ERS/ATS criteria for severe asthma consented to participate in an accelerated treatment schedule (ABT), which treated the whole left lung followed by the right lung four weeks later. The short-term outcomes of these patients were compared with 37 patients treated with conventional BT scheduling (CBT). The outcome measures used to assess safety were (1) the requirement to remain in hospital beyond the electively planned 24-h admission and (2) the need for re-admission for any cause within of 30 days of treatment.

**Results:**

The total number of radiofrequency activations delivered in the ABT group was similar to CBT (187 ± 21 vs 176 ± 40, p = 0.326). With ABT, 11 in 31 admissions (37.9%) required prolonged admission due to wheezing, compared to 5.4% with CBT (p = 0.0025). The mean hospital length of stay with ABT was 1.8 ± 1.3 days, compared to 1.1 ± 0.4 days (p < 0.001). ICU monitoring was required on 5 occasions with ABT (16.1%), compared to 0.9% with CBT (p = 0.002). Subgroup analysis demonstrated that females were more likely to require prolonged admission (OR 11.6, p = 0.0025). The 30-day hospital readmission rate was similar for both groups (6.4% vs 5.4%, p = 0.67). All patients made a complete recovery after treatment with similar outcomes at the 6-month follow-up reassessment.

**Conclusion:**

This study demonstrates that ABT results in greater short-term deterioration in lung function associated with a greater risk of prolonged hospital and ICU stay, predominantly affecting females. Therefore, in females, these risks need to be balanced against the convenience of fewer treatment sessions. In males, it may be an advantage to compress treatment.

## Background

Bronchial thermoplasty (BT) is a bronchoscopic, non-pharmacological intervention for the management of asthma. It offers an alternative therapeutic option for those with severe asthma, defined by the Global Initiative for Asthma (GINA) as those with persistent symptoms requiring step 5 of controller treatment [1]. BT involves the delivery of radiofrequency energy to distal airways of 2–10 mm in diameter, using a catheter electrode introduced by a flexible bronchoscope [2]. The goal of treatment is to induce atrophy in the airway smooth muscle layer, which is known to be hypertrophied in severe asthma [3, 4]. Treatment benefits have been established in three randomised controlled trials, and three real-world registries, which have each demonstrated improved symptom control and quality of life scores, and reduced exacerbation frequency [5–10].

The major adverse effect of bronchial thermoplasty is short-term aggravation of asthma in the immediate post-operative period [2]. Following BT, an average fall in post-bronchodilator Forced Expiratory Volume in 1-s (FEV1) of 9% has been reported [11]. This is maximal 24 h post procedure, after which there is steady recovery. The degree of fall in FEV1 is proportional to the quantum of radiofrequency treatment applied [11]. Historically, BT has been divided into 3 treatment sessions, separated by 3–4 weeks, each treating different portions of the lung [2]. However, this treatment plan does mean that patients require three separate hospital admissions with three separate anaesthetics to complete their treatment. This adds to the cost and the inconvenience of the treatment, particularly when patients live remotely to the treatment centre. If patients could be safely treated in two sessions rather than three, this would be a more attractive proposition for patients, doctors, hospitals and health funds.

Therefore, the aim of this study was to investigate whether BT could be safely compressed into two treatment sessions.

## Methods

### Study subjects

This was a single centre, prospective, observational study conducted at a tertiary referral centre. Patients were evaluated for BT at the request of their treating specialist respiratory physician, having already been evaluated to ensure that (1) comorbidities had been addressed, (2) biological treatments had been instigated where indicated, and (3) adherence with optimized asthma therapy including high dose inhaled corticosteroids and dual long-acting bronchodilator therapy had been demonstrated. All patients were required to meet the European Respiratory Society/American Thoracic Society (ERS/ATS) definition of severe asthma. [12].

During the 18 months, January 2019 to June 2020, 16 patients undertook BT using the accelerated, two-treatment, treatment schedule. The outcomes of these patients were compared with the 37 patients in whom BT had been completed prior to January 2019, where conventional BT scheduling using three treatments had been used. All patients undergoing BT at our centre were included.

### Procedure

Patients being treated in two sessions had the left upper and lower lobes treated in the first treatment session, and then the right upper and lower lobes treated in the second session. As is standard practice, the right middle lobe was not treated. All patients received oral steroid premedication of 50 mg Prednisolone/day for three days prior to the procedure and 3 days post procedure, as with conventional BT. Patients also received inhaled bronchodilators immediately prior to the procedure, and intraoperative intravenous dexamethasone and glycopyrrolate. They were routinely observed in hospital overnight following treatment, with expected discharge the next morning. The number of radiofrequency activations generated at each treatment session was recorded.

### Outcomes

In this study, the primary outcomes related to adverse events, and were defined by (1) admission to hospital beyond the planned 24 h and/or (2) readmission to any hospital for any cause in the 30 days following any treatment session. These events were established by medical record review and by direct patient enquiry. The frequency of adverse events were compared between the accelerated treatment group and the cohort of patients who had received conventional BT. In addition, a calibrated portable spirometer (Jaeger Vyntus Pneumo, Carefusion, Germany) was used to record the post bronchodilator FEV1 immediately preoperatively in theatre, and then again, in the ward 24 h later, in order to quantify the fall in FEV1 post procedure. This data was available for all 16 patients treated with the accelerated treatment plan, but only available for 20 of the 37 patients treated with standard BT.

Secondary outcome measurements related to the therapeutic effects of BT. All patients were evaluated at baseline, 4 weeks prior to the initial BT procedure, by age, gender, BMI, medication history, exacerbation frequency, spirometry and the Asthma Control Questionnaire, 5-item version [13]. Permission to use this instrument had been specifically granted to us by its author, Elizabeth Juniper. Exacerbations were defined by the need for an increase in oral corticosteroids for 3 days. Evaluations were repeated 6 months after the completion of all BT procedures.

Spirometry was undertaken in an accredited laboratory by experienced respiratory scientists, and to ERS/ATS standards, using the Jaeger Vyntus Body (Carefusion, Germany) calibrated on the day of patient testing [14]. Predicted values were drawn from the Global Lung Initiative [15].

### Ethics

This study was prospectively approved by the Peninsula Health Human Research Ethics Committee. Patients were enrolled only after informed consent had been obtained.

### Statistical analysis

For normally distributed data, results are presented as mean ± standard deviation, and comparisons are made with a t-test. Where sample sizes are small, data is presented as median (interquartile range) and comparisons are made with a Wilcoxon signed rank test for paired data, and a Mann–Whitney U test for unpaired data. A Fisher’s Exact test is used to compare categorical data. Statistical significance was taken at p < 0.05 for a two-tailed test.

## Results

### Baseline characteristics

The clinical features of both sets of patients are summarized in Table 1. This was a group of very severe asthmatics, with severely impaired lung function, and high medication and symptom burdens. Time-based differences were evident between the two groups of patients with the more recent BT patients being more severely affected as demonstrated by higher ACQ, higher maintenance dose of oral steroids, and more frequent use of reliever medication. This was expected because many of the conventionally treated patients underwent BT prior to the availability of anti-interleukin-5 monoclonal antibody therapy in Australia (January 2017). As a result, those patients undergoing BT in the latter years, and by the accelerated treatment approach, were more likely to be already being treated with biological therapy, and yet, despite this, still severely symptomatic. (Patients who had done well with biological therapy would not have needed BT).

**Table 1:** Baseline clinical characteristics of BT patients

BT treatment group	Accelerated	Conventional	p
Sample size	16	37	
Age (years)	49.0 (20.8)	59.0 (19.5)	0.154
Males (%)	43.8	48.6	0.660^a^
BMI (kg/m^2^)	34.0 (10.6)	29.0 (7.7)	0.008
Tobacco (pack/years)	0 (7)	0 (9.5)	0.938
ACQ	3.5 (1.5)	3.0 (1.5)	0.259
Exacerbations in 6 months	2.0 (4.4)	2.0 (4.0)	0.868
FEV1 (% predicted)	45.9 (22.1)	49.5 (26.3)	0.291
FER (%)	56.0 (24.25)	51.1 (19.6)	0.106
Change FEV1 post BD (%)	10.9 (29.5)	12.5 (22.5)	0.880
Prednisolone (mg/d)	10.0 (25)	5.0 (10)	0.105
Inhaled steroids (eq/d)	2000 (1000)	2000 (1000)	0.750
SABA (puffs/day)	12.0 (9)	8.0 (14)	0.034
Biological therapy (%)	62.5	13.5	0.001^a^
Bl. Eosinophils (cells/μl)	50 (100)	200 (400)	0.003
IgE (I.U.)	40 (140)	67 (156)	0.330

Median (Interquartile range) p: Mann Whitney test ^a^: Fishers exact test, *BMI* body mass index, *ACQ* Asthma Control Questionnaire, *FEV1* forced expiratory volume 1 s, *FER* forced expiratory ratio, *SABA* short acting beta agonist. Inhaled steroid dose measured in beclomethasone equivalent dose, BD: bronchodilator, I.U: International Units, Bl: Blood

### Treatment

In the accelerated treatment group, 15 patients completed both treatments whilst one patient declined further treatment following the first treatment session. This particular patient was average for the group in terms of baseline FEV1% predicted, ACQ, prednisolone dose and requirement for bronchodilators. However, they were of a particularly anxious predisposition, which the authors believe to be the main reason treatment was not continued. The 37 patients treated with conventional BT completed all 111 treatments.

The total number of radiofrequency activations delivered was similar in both patient groups − 187 ± 21 in the accelerated treatment group, compared to 176 ± 40 in the conventional treatment approach (p = 0.326). Thus, both groups received a similar quantum of treatment independent of the scheduling. In practice, this meant that when the whole left lung was treated in the accelerated treatment group, 100 ± 17 activations were administered in one session, by comparison with 49 ± 14 activations when just the left lower lobe was treated in the conventional approach (p < 0.001). Similarly, on the right side, 89 ± 21 activations were delivered in one session to the right lung in the accelerated protocol, compared with 48 ± 17 activations to the right lower lobe with the conventional approach (p < 0.001). In our centre, we allow 45 min of theatre time for each booked BT case. Although the operating time was 10 min longer when a whole lung was treated by BT, every case was completed within the allowed usual theatre time, and without altering subsequent theatre scheduling.

The mean fall in post bronchodilator FEV1 24 h after BT was 403 ± 352 ml, or 22.6 ± 16.4% with accelerated treatment. This was significantly greater than when either the right or left lower lobes were treated with conventional scheduling, where the fall after treatment in FEV1 was 114 ± 243 ml or 5.0 ± 15.0% (p = 0.001). However, the fall in FEV1 after conventional upper lobe treatment was not statistically different from treating either the whole left or right lung (p = 0.203) (Table 2).

**Table 2:** Change in post bronchodilator FEV1 24 h post BT

	n	Activations	FEV1 day 0	FEV1 day 1	% fall	p
Accelerated group						
Left lung	16	100 ± 17	1.80 ± 1.01	1.45 ± 0.95	21.3 ± 13.5	0.001
Right lung		89 ± 21	1.80 ± 0.91	1.35 ± 0.74	23.9 ± 19.8	0.001
Conventional group						
Left lower lobe	20	49 ± 14	1.63 ± 0.58	1.54 ± 0.52	3.5 ± 16.2	0.140
Right lower lobe		48 ± 17	1.64 ± 0.56	1.50 ± 0.47	6.6 ± 13.9	0.010
Upper lobes		84 ± 37	1.70 ± 0.58	1.43 ± 0.57	17.1 ± 12.6	0.001

Mean ± standard deviation p compares the FEV1 on day 1 with day 0 by paired t test

FEV1: Forced Expiratory Volume in 1-s (litre)

### Adverse events

Patients remaining in hospital longer than 24 h after the procedure were deemed to have experienced an adverse event, and with standard treatment, this occurred in 6 instances of 111 admissions (5.4%). By comparison, with accelerated treatment, there were 11 occurrences in 31 admissions (37.9%) when patients remained in hospital after 24 h (p < 0.001). The medical notes recorded that in each case this was due to wheezing. The mean hospital length of stay for patients receiving accelerated treatment was 1.8 ± 1.3 days, compared to 1.1 ± 0.4 days in the standard group (p < 0.001). No patient required endotracheal intubation and mechanical ventilation, but there were 5 occasions in the accelerated treatment group (16.1%) when patients required monitoring in the Intensive Care Unit, compared to 1 occasion (0.9%) in the standard treatment group (p = 0.002).

Two patients (6.4%) in the accelerated treatment group were readmitted within 30 days of a BT procedure, one for pneumonia and one for non-ischaemic chest pain. Both made a full recovery. This readmission rate was similar in the standard treatment group (5.4%, p = 0.670).

In the accelerated treatment group, a subgroup analysis was conducted to compare the baseline characteristics of those patients who remained in hospital longer than 24 h with those who were discharged within 24 h as originally planned. These results are shown in Table 3. Across most parameters, there were no distinguishing differences. However, there appeared to be a gender difference. For males, there was a 1 in 14 admissions chance (7.1%) of remaining in hospital after 24 h with accelerated treatment, whilst in females this chance was 6 in 17 admissions (35.2%) (p = 0.090). Interestingly, in our standard treatment group, all 6 instances of prolonged admission were also all females. Hence, for the pooled group of 53 patients undergoing 142 BT procedures, there was one male admission (1.4%) and 12 female admissions (16.2%) longer than 24 h, resulting in an odds ratio for prolonged hospital stay of 11.6 females to males (p = 0.0025).

**Table 3:** Clinical characteristics of patients treated with accelerated BT protocol and remaining in hospital longer than 24 h post BT

	Hospital stay ≤ 24 h	Hospital stay > 24 h	p
n	9	7	
Age (years)	50.0 (20)	48 (25)	0.918
Male: Female	6:3	1:6	0.060^a^
BMI (kg/m^2^)	34 (11)	36 (13)	0.210
ACQ	3.2 (1.6)	3.6 (1.6)	0.918
FEV1 (% predicted)	45.7 (22.8)	56.5 (15.9)	0.681
Exacerbations (6 months)	2 (5)	4 (5)	1.000
Prednisolone (mg/d)	0 (25)	20 (30)	0.210
Inhaled Steroids (eq/d)	1600 (1000)	2000 (2000)	0.351
SABA (puffs/d)	12 (18.5)	12 (6)	0.837
Activations	180 (49)	172 (36)	0.272

Median (Interquartile range), p: Mann–Whitney U test ^a^: Fisher’s exact test. *BMI* body mass index, *ACQ* Asthma Control Questionnaire, *FEV1* forced expiratory volume 1 s, *SABA* short acting beta agonist. Inhaled steroid dose measured in beclomethasone equivalent dose

To explore why there was a higher adverse event rate in females, two further comparisons were made. The baseline characteristics of the 28 females were compared with 25 males (Table 4). Overall, both groups of patients were found to be very similar, but, as expected, males had larger lungs. Therefore, the fall in lung capacity following BT treatment was compared by gender for the pooled group of 36 patients (19 female, 17 male) where FEV1 had been measured routinely 24 h post procedure. This comparison is shown in Table 5. The data suggests that the percentage fall in FEV1 post BT is significantly less in males, who are protected by higher baseline lung volumes.

**Table 4:** Clinical characteristics by gender

	Females	Males	p
n	28	25	
Age (years)	55.2 ± 11.1	56.8 ± 15.4	0.670
BMI (kg/m^2^)	31.5 ± 7.8	30.2 ± 6.5	0.516
ACQ	3.5 ± 0.9	3.2 ± 1.1	0.255
FEV1 (%predicted)	53.6 ± 19.4	48.4 ± 13.6	0.271
FEV1 (litre)	1.29 ± 0.56	1.70 ± 0.65	0.017
Exacerbations (6 m)	3 (4)	2 (3.5)	0.245 ^a^
Prednisolone (mg/d)	10.2 ± 12.5	8.4 ± 13.2	0.603
Inhaled Steroids (eq/d)	1675 ± 900	1796 ± 852	0.618
SABA (puffs/d)	11.5 (13)	6 (9)	0.046 ^a^
Bl eosinophils (cells/μl)	228 ± 246	262 ± 318	0.647
IgE (IU/ml)	50 (179)	52 (312)	0.286^a^
Total activations	175 ± 36	183 ± 38	0.409

Mean ± standard deviation, median (Interquartile range) p: t-test ^a^: Mann Whitney U test

*BMI* body mass index, *ACQ* Asthma control Questionnaire, *FEV1* forced expiratory volume 1-s, *SABA* short acting beta agonist Inhaled steroids; beclomethasone equivalent dose in micrograms

**Table 5:** Change in FEV1 post procedure by gender

	Females	Males	p
n	19	17	
Post BD FEV1 day 0 (litre)	1.49 ± 0.57	1.86 ± 0.78	0.015
Post BD FEV1 day 1 (litre)	1.22 ± 0.50	1.65 ± 0.67	0.001
Volume change (litre)	0.27 ± 0.28	0.21 ± 0.32	0.376
% change from baseline	− 17.2 ± 18.0	− 9.5 ± 15.9	0.034

p: t test FEV1: Forced Expiratory Volume 1 s BD: bronchodilator

The accelerated treatment group were more obese than their conventional comparitors (Table 1). To ensure that the higher adverse event rate was not an effect of obesity, the baseline BMI was compared in the 10 patients who stayed in hospital longer than 24 h (33.9 ± 2.0) with the 43 patients who were discharged within 24 h (30.2 ± 7.3), and this difference was not statistically significant (p = 0.14).

### Outcomes 6 months post procedure

The clinical responses to treatment were measured 6 months after the completion of BT, and these results are presented in Table 6. Substantive, clinically meaningful improvements were observed in ACQ, exacerbation frequency, and medication usage. A trend towards improvement in FEV1 was observed. The magnitude of the changes were similar, and not statistically different, in both treatment groups. For example, the mean improvement in ACQ was 1.2 ± 1.2 in the accelerated treatment group, compared to 1.4 ± 1.3 with conventional teeatment (p = 0.56), and the median (Q1, Q3) improvement in short acting beta-2 agonist use was − 7.0 (− 12, − 3.5) puffs/day in the accelerated treatment group compared to − 4.0 (− 9.0) puffs/day with conventional treatment (p = 0.19).

**Table 6:** Clinical outcomes following BT

	Pre BT	6 months post BT	p
Accelerated treatment (n = 15)			
ACQ	3.5 (1.5)	2.4 (1.6)	0.005
SABA (puffs/day)	12.0 (9)	6.0 (8.5)	0.006
Exacerbations per 6 months	2.0 (4.4)	0 (2.0)	0.007
Prednisolone (mg/day)	10.0 (25)	7.5 (10)	0.058
FEV1 (% predicted)	45.9 (22.1)	51.6 (40.6)	0.008
Conventional treatment (n = 37)			
ACQ	3.0 (1.5)	1.6 (1.7)	0.001
SABA (puffs/day)	8.0 (14.0)	1.0 (6.25)	0.001
Exacerbations per 6 months	2.0 (4.0)	0.0 (1.5)	0.001
Prednisolone (mg/day)	5.0 (10)	0 (7.5)	0.015
FEV1 (% predicted)	49.5 (23.2)	51.6 (22.8)	0.248

p = Wilcoxon signed rank test ACQ: Asthma Control Questionnaire, *SABA* short acting beta agonist, *FEV1* forced expiratory volume 1 s

## Discussion

This is the first study to examine the delivery of BT in two treatment sessions, and make comparisons with conventional treatment in three sessions. Whilst both groups of patients experienced favourable and comparable outcomes at six months, a higher prevalence of prolonged admission was observed in the accelerated group immediately post-procedure (37.9% vs 5.4%). The implications of this will be explored.

This was a cohort of patients with very severe asthma. They had a high symptom burden despite biological therapies and oral steroids, and the mean FEV1 of 45.9% predicted was considerably lower than participants in the AIR and AIR 2 trials (whose participants had an FEV1 of > 60% predicted), and RISA of > 50% predicted [16–18]. It is common for asthma symptoms to initially worsen as the result of acute airway inflammation and oedema from BT [19]. Those in the accelerated group had a larger number of airways treated in each session, and previous studies have demonstrated a relationship between higher activations delivered and a greater decline in FEV1 [11]. This finding is supported by the greater decline in FEV1 observed 24 h post-procedure with the accelerated treatment in this study. Therefore, it is not surprising that these patients took longer to recover. Nevertheless, the effects seen in the accelerated group were transient. The mean hospital stay was 1.8 ± 1.3 days, and, following hospital discharge, the readmission rate was low and similar between the two treatment groups.

The subgroup analysis suggests that the risk of prolonged hospital admission following BT pertained predominantly to females, and this occurred irrespective of the treatment regimen delivered. This has not been previously noted. The three randomized controlled trials of BT did not present a breakdown of adverse effects by gender. The odds ratio of 11.6 for prolonged hospital stay in females is so strong in this study it seems unlikely to have occurred by chance. Table 4 demonstrates the similarity in clinical characteristics here between the females and males, save for the expected anthropomorphic difference of a lower absolute FEV1 in females. We postulate that this may be a factor in the higher post-operative adverse event rate in females. The volume change in FEV1 after BT appears to be at least as great in females as males, but with females starting from a lower baseline, the impact of the deterioration becomes substantially greater (Table 5).

There are attractions to performing BT in fewer sessions. Hospital admission and post-operative recovery are disruptive to a patient’s life. Patients in this study were generally enthusiastic about the concept of compressing treatment into two sessions, even when informed of a potential risk of a longer hospital stay. This became a particularly strong advantage for patients who lived interstate or remotely to the treatment centre. Some patients also felt it was a significant advantage in having two anaesthetics rather than three. This study demonstrates that there is obviously a trade-off between the convenience of two treatments and the inconvenience of greater short-term post-operative deterioration.

There are also differing economic implications depending on the drivers in health services. In a country where access to theatre is a major constraint in the delivery of surgery, such as in a publicly funded healthcare system, the ability to perform BT in two treatments is a significant step forward, and would reduce surgical wait times. On the other hand, in areas where the high cost of an overnight hospital bed is the predominant driver in healthcare delivery, such as the United States of America, bronchoscopists may be better to continue to offer traditional 3 session BT on a day-case basis. In such a country, an alternative approach to improve patient convenience, may be to offer single treatment, limited BT, targeted by preprocedural hyperpolarized Magnetic Resonance Imaging (MRI) [20]. This technique shows great promise, but is currently severely limited by the lack of availability of hyperpolarized MRI in most centres.

Being a feasibility study, the numbers of patients studied here were deliberately small, as we were concentrating on establishing patient safety in the first instance. The technique would need repeating on a larger scale before firm recommendations could be made. We must acknowledge that this study was non-randomized, and that time dependent differences between the two patient groups are evident. However, given that the accelerated treatment group were a more severe group of asthmatics, this would serve to exaggerate any differences in safety between the two techniques rather than provide false reassurance. In that sense, this is unlikely to be a limitation and the results presented are more akin to a worst-case scenario.

This study shows that it is possible to compress BT into two treatments, and it appears particularly safe to do so in males. However, there is a penalty to pay by taking this approach, namely a greater fall in FEV1 in the immediate postoperative period. Therefore, at our centre, we are not offering this approach to those patients whose baseline FEV1 is less than 50% predicted, until further data becomes available.

Improving and refining treatment procedures to minimise patient discomfort and maximise efficiency is a natural development in the evolution of any medical procedure. Further research on a larger scale is required to confirm our results, but accelerating the delivery of BT appears to be safe in some patients without compromising clinical outcomes.

## Data Availability

The datasets used during the current study are available from the corresponding author on reasonable request.

## Abbreviations

BT: Bronchial thermoplasty
FEV1: Forced expiratory volume in 1 s
VC: Vital capacity
FER: Forced expiratory ratio
ERS/ATS: European Respiratory Society/American Thoracic Society
ACQ: Asthma Control Questionnaire (5-item version)
SABA: Short acting beta-agonist
MRI: Magnetic Resonance Imaging
